# Reproductive health status and related knowledge among women aged 20–39 years in rural China: a cross-sectional study

**DOI:** 10.1186/s12978-020-00939-2

**Published:** 2020-06-10

**Authors:** Mingzhu Chen, Yang Luo, Jingxia Fu, Ting Wang, Yanting Meng, Chen Xu, Si Qin

**Affiliations:** Xiang Ya Nursing School, Central South University, Changsha, 410013 China

**Keywords:** Reproductive health status, Knowledge, Rural women, China

## Abstract

**Background:**

Reproductive health is the core science of human life and is critical to the healthy and sustainable development of human society. Since 1980, China has enforced a “one child” policy. With the implementation of the Universal Two-Child Policy in 2016, every couple is allowed to have two children instead of one, which will lead to more pregnancies, births, and advanced maternal age. Thus, women aged 20–39 years, at the peak of sexual activity and fertility, will face more reproductive health problems related to pregnancies and births. This study aimed to investigate the current reproductive health status, knowledge, and factors associated with reproductive health knowledge among women aged 20–39 years in rural China.

**Methods:**

A cross-sectional study was conducted in five villages of five cities in China. The data were collected using pre-tested and structured questionnaires through face-to-face interviews. The data were entered into Epidata version 3.0, and analyzed using SPSS version 18.0. A descriptive summary of the data and logistic regression were used to identify associated factors.

**Results:**

One-third of the participants reported that they had suffered from gynecopathy, and 38.89% of participants with gynecopathy-related discomfort did not seek medical treatment. Condoms and intrauterine devices were the main contraceptive measures used, and 28.70% of women had a history of induced abortion. Over half of the respondents (53.00%) were classified as having a low reproductive health knowledge score. Factors associated with lower knowledge levels were lower education, no history of gynecopathy, and lack of acquiring knowledge from medical staff, WeChat/micro-blog, or the internet.

**Conclusion:**

A poor reproductive health situation and low level of health knowledge were found among women aged 20–39 years in rural China. More specific interventions promoting reproductive health and targeting rural women aged 20–39 years are needed.

## Plain English summary

There is an increasing concern about reproductive health problems in developing countries. With the opening of China’s Universal Two-Child Policy, women aged 20–39 years, at the peak of sexual activity and fertility, will face more reproductive health problems related to pregnancy, especially in less-developed rural areas.

In order to address the issue, this study investigated the reproductive health status and knowledge of women aged 20–39 years via survey and interview in rural China. Among the 973 participants, one-third had suffered from gynecopathy. Condoms and intrauterine devices were the main contraceptive measures used, and the rate of induced abortion was 28.70%. Over half of the respondents (53.00%) had a low reproductive health knowledge score. Factors associated with lower knowledge levels were lower education, no history of gynecopathy, and lack of acquiring knowledge from medical staff, WeChat/micro-blog, or the internet.

In conclusion, there was a poor reproductive health situation and low level of reproductive health knowledge among women aged 20–39 years in rural China. This finding suggests that there is a need to focus on specific interventions and reproductive health education for women aged 20–39 years in rural China.

## Background

Reproductive health (RH) is a concept first proposed by the World Health Organization (WHO) Special Programme on Human Reproductive Research in 1988 and finalized in 1994. RH refers to the physical state of the reproductive system and functions, as well as the mental and social adaptations in the reproductive process [1]. At the 4th International Conference on Population and Development in Cairo, WHO proposed the global goal of “reproductive health for all by 2015” [2], and the conference brought RH to the forefront of world attention. In 2010, the Outline for the Development of Chinese Women (2010–2020) mentioned “improving women’s reproductive health and ensuring women’s access to reproductive health technology services” as a goal of national and social development [3]. RH is a core science of human life and is critical to the healthy and sustainable development of human society [4].

Family planning has been a national priority in China since the 1970s, especially since 1980 when the “one-child” policy began. This policy restricted urban couples to one child and some rural couples, whose first child was a girl, to two children [5]. However, in 2016, the Chinese government implemented the Universal Two-Child Policy, allowing each couple to have two children. With this policy, the number of Chinese women choosing to have children may increase sharply in a short span of time, and RH problems related to pregnancy and childbirth will become more prominent. This is particularly significant for women aged 20–39 years, who are in a peak sexual and reproductive period. Several studies in China have shown that women aged 20–39 years are at high risk of sexually transmitted infections (STIs) and reproductive tract infections (RTIs), and also represent the majority of multiple abortions [6–8]. Thus, the RH of these women needs to be an area of focus.

In China, due to the privacy of RH and the constraints of traditional conservative ideas, rural women have little awareness of their own RH or health care. Most of them have not received RH education and have limited knowledge [9, 10]. Health knowledge is key to enabling women to be aware of their health status and to seek appropriate health services [11]. The 2007 China State Council Working Committee on Women and Children report showed that RTI and STI rates were significantly higher in rural women than in urban women [12]. Monoarul et al. also found a wide gap in reproductive behaviors and health knowledge between urban and rural women [13].

Although some previous studies have described the RH status and related knowledge of women in China, the situation of rural women aged 20–39 years is still largely unknown. The aim of this study was to investigate RH status, knowledge, and factors associated with RH knowledge among women aged 20–39 years in rural China, to provide a basis for further interventions to improve RH.

**Fig. 1:**
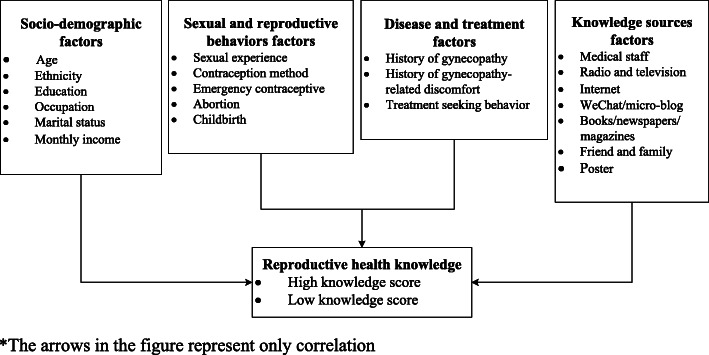
A logical framework for the study of RH knowledge among women aged 20–39 years in rural China

On the basis of extensive literature research, our logical framework (Fig. 1) identifies RH knowledge as an outcome variable which is associated with a range of factors, including socio-demographic, sexual and reproductive behaviors, disease and treatment, knowledge sources factors [12, 14, 15].

## Methods

### Design and sample

This cross-sectional study was conducted in Hunan Province in cooperation with the Women’s Federation of Hunan Provincial Government between April 1 and August 20, 2018. Hunan Province in south-central China has a population of 68.6 million. We selected one county each in the east, west, south, north, and central areas of Hunan Province that we believed to be typical of each region, then selected one city in each county. Next, multistage cluster sampling was used to recruit samples from these five selected cities. We randomly selected one township per city, and then one village from each township, for a total of five villages. Finally, 1000 women who met the age requirement (20–39 years) were recruited.

### Data collection

Investigators received training, conducted interviews with the eligible subjects, and explained the purpose and procedures of the study. The questionnaires, which were anonymous, were either completed by the subjects themselves or with the help of the investigators in cases of subjects with limited literacy.

### Measurements

The investigators designed a Women’s Reproductive Health Questionnaire (WRHQ) that was developed after extensive literature research and evaluated by a panel of 10 experts from Central South University, affiliated hospitals, and Hunan Women’s Federation. The questionnaire contained four components, including socio-demographic characteristics (age, ethnicity, education, occupation, marital status and average monthly household income); sexual and reproductive behaviors (sexual experience, last contraceptive methods used, emergency contraceptive use, and the number of abortions and children); gynecopathy and medical treatment (history of gynecopathy, gynecopathy-related discomfort, and treatment or reason for non-treatment); and RH knowledge, with a total of 30 items.

Fifteen RH knowledge questions were included in the questionnaire: three concerning RTIs and STIs, six regarding contraception and induced abortion, and six about cervical cancer and HPV vaccine. These included eight single-choice questions and seven multiple-choice questions. Each single-choice question was credited with a score of one for a correct response and zero for incorrect or indecisive responses, with a maximum single-choice question score of 8. Due to the low rate of correct answers to multiple-choice questions, each correct option in each multiple-choice question was credited with a score of one and incorrect option with a score of zero. There were 37 total correct options for multiple-choice questions. The single and multiple-choice values were totaled to obtain a knowledge score, with a maximum score of 45. We divided the responses into low score (≤ median score) and high score groups (> median score). In addition, participants were asked how they acquired RH knowledge.

### Operational definitions

#### Induced abortion

Induced abortion refers to the termination of pregnancy by artificial method due to unexpected pregnancy, disease and other reasons [16].

#### Gynecopathy

Gynecopathy refers to diseases of the female reproductive system, including diseases of the vulva, vagina, uterus, fallopian tubes and ovaries [16].

### Data analysis

All data were independently double-entered and validated using EpiData (Version 3.0., The Epidata Association, Odense, Denmark). Data were analyzed with SPSS 18.0 (Version 18.0., Chicago: SPSS Inc.). Characteristics were summarized with counts (percentages) for categorical variables, and median (standard deviation, SD) for continuous variables. Both bivariate and multivariable logistic regression analyses were done to identify factors associated with RH knowledge level among rural women. Bivariate logistic regression analysis was carried out to compare RH knowledge level among different subgroups. Variables with a p-value of < 0.05 in the bivariate analysis were further entered into the final multivariate logistic regression model. Multivariable logistic regression analysis was performed to adjust for possible confounding variables. Crude and adjusted odds ratios with their 95% confidence intervals were calculated. The strength of the statistical association was assessed by odds ratios (OR) with 95% confidence intervals. In the multivariable analysis, a variable with a *p*-value of < 0.05 was considered statistically significant. If more than 20% of the items had missing values, the questionnaire would be excluded as invalid.

### Ethical considerations

The study was approved by the Ethical Committee of the Xiangya Nursing School, Central South University. Written consent was obtained from all respondents before the interview.

## Results

### Socio-demographic characteristics

A total of 1000 women who met the entry requirements were recruited in this study, and 973 (97.3%) participants completed the questionnaire as requested. Almost all participants (97.7%) were ethnic Han, 86.0% were married, and the mean age was 29.41 (SD = 5.69) years. There were only 21.7% of women with college education or above. More than half (53.9%) were farmers or workers. Average monthly household income was CNY 3000–4999 for 33.3% and below CNY 3000 for 29.3% (Table 1).

**Table 1:** Socio-demographic characteristics of rural women aged 20–39 years, Hunan, China, 2018 (*n* = 973)

Characteristics	n	%
**Age (years)**		
20–25	283	29.1
26–30	253	26.0
31–35	251	25.8
36–39	186	19.1
**Ethnicity**		
Ethnic Han	951	97.7
Ethnic minority	9	0.9
**Education**		
Senior high school or less	749	77.0
College or more	211	21.7
**Occupation**		
Students	49	5.0
Farmers or workers	524	53.9
Civil servants/administrators	29	3.0
Professional technicians	69	7.1
Business/service personnel	122	12.5
Unemployed	152	15.6
**Marital status**		
Married	837	86.0
Unmarried	135	13.9
**Average monthly household income (CNY**^**a**^**)**		
<¥3000	285	29.3
¥3000–4999	324	33.3
¥5000–7999	257	26.4
≥ ¥8000	98	9.6

^a^CNY: Chinese Yuan; 6.704 CNY = 1 USD (14 April 2019)

### Sexual and reproductive behaviors

Of the 817 (84.0%) participants with sexual experience, 68.3% were currently using contraceptives, and nearly one-third had used emergency contraceptives. Condoms (52.0%) were the most frequently used contraception, followed by intrauterine devices (IUD, 29.9%). More than 20% had a history of induced abortion and 80.1% had at least one child (Table 2).

**Table 2:** Sexual and reproductive behaviors information of rural women aged 20–39 years, Hunan, China, 2018 (n=973)

Characteristics	n	%
**Having sexual experience**		
Yes	817	84.0
No	135	13.9
**Current contraceptive use (*n*** **= 817)**		
Yes	558	68.3
No	271	33.2
**Use of emergency contraceptives (*n*** **= 817)**		
Yes	230	28.2
No	587	71.8
**Contraceptive methods for the recent sexual intercourse (*n*** **= 558)**		
Condom	290	52.0
Oral contraceptives (OC)	30	5.4
Coitus interruptus	31	5.6
Contraception during safe period	66	11.8
Intrauterine device (IUD)	167	29.9
Emergency contraceptives	6	1.1
Ligation	67	12.0
Contraceptive film / ointment	2	0.4
Rorplant	0	0.0
External use sperm killing agent	1	0.2
**Number of induced abortions**		
0	758	77.9
1	133	13.7
≥ 2	82	8.4
**Number of children**		
0	194	19.9
1	359	36.9
≥ 2	420	43.2

### Gynecopathy and medical treatment seeking behavior

One third (33.6%) of participants reported suffering from gynecopathy. Colpitis (inflammation of vaginal tissue or vaginitis) (24.2%) was the most common gynecological disease. Most participants (81.5%) reported no gynecopathy-related discomfort in the last 2 weeks. Nearly 40% of the participants with gynecopathy-related discomfort did not seek medical treatment, primarily because they did not think it was necessary (Table 3).

**Table 3:** Gynecopathy-related information of rural women aged 20–39 years, Hunan, China, 2018 (*n* = 973)

Characteristics	n	%
**History of gynecopathy**		
No	646	66.4
Colpitis	235	24.2
Cervicitis	59	6.1
Pelvic infection	87	8.9
Hysteromyoma/adenomyosis	11	1.1
Oophoritic cyst	15	1.5
Endometriosis	1	0.1
Gynecologic tumor	1	0.1
Pelvic floor dysfunction	2	0.2
Abnormal menstruation	56	5.8
Other	4	0.4
**Discomfort in the past two weeks**		
No	793	81.5
Leucorrhea abnormality	103	10.6
Pruritus or burning of vulva	30	3.1
Lumbar and abdominal pain	31	3.2
Sexual pain or vaginal bleeding	6	0.6
Frequent urination and urgency	20	2.1
Irregular menstruation	53	5.5
Other	9	0.9
**Whether to seek medical assistance after discomfort (*n*** **= 180)**		
Yes	110	61.1
No	70	38.9
**Why not to seek medical treatment**		
It’s not necessary. Just carry it.	30	42.9
Embarrassed	8	11.4
Self-medication	13	18.6
To save money	3	4.3
No effectively handled	2	2.9
Having no time	12	17.1
Other	2	2.9

### RH knowledge sources

RH knowledge was most commonly obtained from medical staff (59.0%) and the internet (47.9%). Posters (12.2%) and books/newspapers/magazines (19.1%) had less impact (Table 4).

**Table 4:** Reproductive health (RH) knowledge sources of rural women aged 20–39 years, Hunan, China, 2018 (*n* = 973)

Characteristics	n	%
**Did you acquire knowledge from medical staff**		
Yes	574	59.0
No	386	39.7
**Did you acquire knowledge from radio and television**		
Yes	285	29.3
No	676	69.5
**Did you acquire knowledge from the internet**		
Yes	466	47.9
No	495	50.9
**Did you acquire knowledge from WeChat/micro-blog**		
Yes	346	35.6
No	615	63.2
**Did you acquire knowledge from books/newspapers/magazines**		
Yes	186	19.1
No	775	79.7
**Did you acquire knowledge from friend and family**		
Yes	275	28.3
No	686	70.5
**Did you acquire knowledge from poster**		
Yes	119	12.2
No	842	86.5

### RH knowledge level and associated factors

The median total score for the participants was 13.00 (SD = 8.80) out of a maximum of 45 points. Over half (516, 53.0%) were classified as having a low RH knowledge score (≤ median score). Bivariate logistic regression was used to compare the RH knowledge level among the variable subgroups. There were statistically significant differences in knowledge level by education, occupation, marital status, average monthly household income, sexual experience, use of emergency contraceptives, number of children, history of gynecopathy, discomfort in the past 2 weeks, and acquiring knowledge from medical staff, radio and television, the internet, WeChat/micro-blog, books/newspapers/magazines, or poster (all *p* < 0.05; Table 5).

**Table 5:** Bivariate and multivariable logistic regression analyses for factors associated with RH knowledge level among rural women aged 20–39 years, Hunan, China, 2018 (*n* = 973)

Characteristics	RH knowledge level f (%)		COR (95%CI)	AOR (95%CI)
	Low	High		
**Education**				
Senior high school or less	419 (55.9)	330 (44.1)	1.36 (1.18–1.57)*	1.40 (1.13–1.74)*
College or more	91 (43.1)	120 (52.9)	1.0	1.0
**Occupation**				
Students	29 (60.1)	20 (39.9)	0.73 (0.54–0.99)*	1.02 (0.45–2.50)
Farmers or workers	311 (59.4)	213 (40.7)	0.57 (0.40–0.82)*	0.65 (0.42–1.01)
Civil servants/administrators	10 (34.5)	19 (65.5)	1.58 (0.69–3.62)	0.47 (0.15–1.47)
Professional technicians	15 (21.7)	54 (78.3)	2.99 (1.55–5.76)*	3.53 (0.98–8.87)
Business/service personnel	54 (44.3)	68 (55.7)	1.05 (0.65–1.69)	0.85 (0.47–1.52)
Unemployed	69 (45.4)	83 (54.6)	1.0	1.0
**Marital status**				
Married	427 (51.0)	410 (49.0)	0.56 (0.38–0.81)*	0.93 (0.36–2.43)
Unmarried	88 (65.2)	47 (34.8)	1.0	1.0
**Average monthly household income (CNY)**				
<¥3000	179 (62.8)	106 (37.2)	0.41 (0.26–0.65)*	0.81 (0.43–1.52)
¥3000–4999	159 (49.1)	165 (50.9)	0.72 (0.45–1.13)	1.20 (0.65–2.22)
¥5000–7999	132 (51.4)	125 (48.6)	0.65 (0.41–1.05)	1.11 (0.59–2.08)
≥ ¥8000	40 (40.8)	58 (59.2)	1.0	1.0
**Having sexual experience**				
Yes	410 (50.2)	407 (49.8)	0.50 (0.34–0.74)*	1.35 (0.57–3.19)
No	90 (66.7)	45 (33.3)	1.0	1.0
**Use of emergency contraceptives**				
Yes	86 (37.4)	144 (62.6)	0.53 (0.42–0.80)*	0.80 (0.50–1.30)
No	430 (57.9)	313 (42.1)	1.0	1.0
**Number of children**				
0	124 (63.9)	70 (36.1)	0.57 (0.40–0.81)*	0.59 (0.29–1.17)
1	181 (50.4)	178 (49.6)	0.99 (0.75–1.32)	0.93 (0.64–1.33)
≥ 2	211 (50.2)	209 (49.8)	1.0	1.0
**No history of gynecopathy**				
Yes	383 (59.3)	263 (40.7)	2.21 (1.68–2.91)*	2.06 (1.39–3.04)*
No	127 (39.7)	193 (60.3)	1.0	1.0
**No discomfort in the past two weeks**				
Yes	435 (54.9)	358 (45.2)	1.63 (1.17–2.28)*	1.49 (0.92–2.41)
No	73 (42.7)	98 (57.3)	1.0	1.0
**Did you acquire knowledge from medical staff**				
Yes	243 (42.3)	331 (57.7)	0.34 (0.26–0.45)*	0.26 (0.15–0.44)*
No	264 (68.4)	122 (31.6)	1.0	1.0
**Did you acquire knowledge from radio and television**				
Yes	124 (43.5)	161 (56.5)	0.59 (0.45–0.78)*	0.90 (0.52–1.81)
No	383 (56.7)	293 (43.3)	1.0	1.0
**Did you acquire knowledge from internet**				
Yes	207 (44.4)	259 (55.6)	0.52 (0.40–0.67)*	0.50 (0.30–0.83)*
No	300 (60.6)	195 (39.4)	1.0	1.0
**Did you acquire knowledge from WeChat/micro-blog**				
Yes	129 (37.3)	217 (62.7)	0.37 (0.28–0.49)*	0.26 (0.15–0.45)*
No	378 (61.5)	237 (38.5)	1.0	1.0
**Did you acquire knowledge from books, newspapers, magazines**				
Yes	74 (39.8)	112 (60.2)	0.52 (0.38–0.72)*	1.04 (0.52–1.68)
No	433 (55.9)	342 (44.1)	1.0	1.0
**Did you acquire knowledge from poster**				
Yes	30 (25.2)	89 (74.8)	0.26 (0.17–0.40)*	0.63 (0.41–1.02)
No	477 (56.7)	365 (43.4)	1.0	1.0

*p < 0.05

*The arrows in the figure represent only correlation

**Fig. 2:**
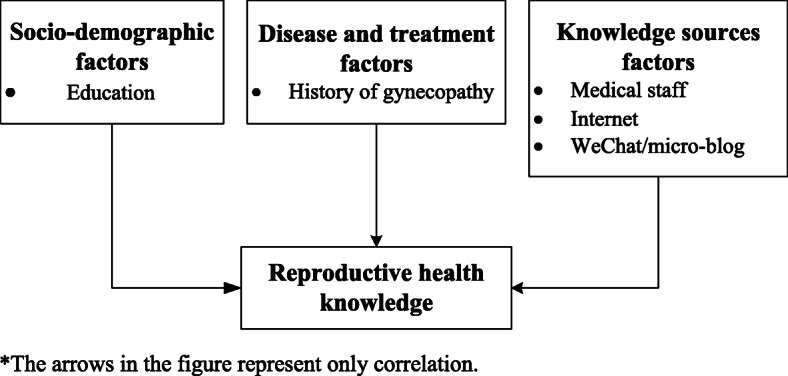
Factors associated with RH knowledge level

Multivariate analysis was used to identify the factors associated with RH knowledge level. RH knowledge level was a dependent variable and variables with statistical significance in the bivariate logistic regression analysis were independent variables. A low knowledge score was assigned as 1, and a high knowledge score was assigned as 0. The multivariable regression model revealed that five variables were associated with RH knowledge level: education, history of gynecopathy, and acquiring knowledge from medical staff or from WeChat/micro-blog or internet (Table 5). Lower education, no history of gynecopathy, and lack of acquiring knowledge from medical staff, WeChat/micro-blog, or internet were all associated with lower RH knowledge levels (Fig. 2).

## Discussion

This study suggests that there is a poor RH situation and low level of RH knowledge among women aged 20–39 years in rural China. Factors associated with knowledge level were education, history of gynecopathy, and acquiring knowledge by medical staff, WeChat/micro-blog, or the internet.

This study showed that 22.1% of women had a history of induced abortion, which is lower than the rates reported in other studies in China [9, 17]. One possible explanation is the 2016 initiation of the Universal Two-Child Policy. The prevalence of induced abortion in China is always higher than that in other developing countries [18, 19], possibly due to different local laws, social and cultural attitudes towards fertility control, and the roles of women in society. The legitimacy of induced abortion, the decrease of fertility intention, and misleading advertisements which lead women into thinking abortion is simple, safe, and free of complications will all cause these rates to remain high. Moreover, Zhang and Wu [20] have shown that a large number of induced abortions and repeat abortions in China are also due to contraceptive failure or lack of contraception.

The primary contraceptive measures reported in this research were condoms and IUDs, consistent with previous studies in China [21, 22]. Nearly 20% of participants, however, were still using coitus interruptus and safe (non-ovulation) period intercourse for contraception. The use of new effective methods, such as oral contraceptives (OC) (5.38%) and contraceptive implants (0), was very low. Ineffective contraceptives and misuse of contraceptives both lead to contraceptive failure and increase the probability of induced abortions resulting from unwanted pregnancies. In China, basic education is deeply influenced by traditional concepts. Most educators and parents are reticent to discuss sex in physical health education, so the quality of sexual education is far from adequate. Future emphasis should be placed on increasing sexual education, including contraception knowledge, from hospitals, communities, and schools.

We found that 33.61% of participants had a history of gynecopathy, which was below rates reported in other studies [23, 24] in rural China, especially for the prevalence of colpitis and cervicitis. In recent years, China’s rural medical health has significantly improved, and a number of government projects aimed at rural women’s health have achieved certain results. However, another probable reason for the discrepancy in gynecopathy rates is the information-gathering method used in this study. Self-reported status has been shown to be a poor measure of prevalence in rural China and elsewhere [14, 25], because it represents only the perceived problems. In addition, lower educational level, backward feudal ideology, and the privacy of gynecological diseases may have made our participants hide the history of gynecopathy, because they often think that gynecological diseases are related to sexual misconduct.

Our data showed that most (81.50%) participants reported no reproductive system-related discomfort in the past 2 weeks. Among women with discomfort, 38.89% did not seek medical treatment, primarily because they considered it unnecessary. This finding was consistent with previous studies conducted in China [26], which suggested that the healthcare use rate of rural women is low. The lack of self-care awareness caused by low education level and constraints caused by low income level contribute to the choice not to seek medical treatment. In the future, governments need to pay more attention to advertising and education, as well as free screening and treatment of common gynecological diseases for women in rural areas.

We also discovered that the RH knowledge level of rural women aged 20–39 years was generally low, as demonstrated by the median questionnaire score of 13 points, or only 28.88% of the full score (45 points). The largest number of participants chose medical staff to acquire medical knowledge, followed by use of the internet. However, a previous study reported that RH knowledge in this population mainly came from parents, friends, and books, although the optimal source was medical staff [27]. The change in knowledge acquisition is due to the rise of township medical services and the progress of science and technology in recent years. Rural women’s access to diagnosis and treatment by medical staff has greatly increased, and at the same time, the internet and mobile phones have spread to every household.

The present study indicated that lower education, no history of gynecopathy, and lack of acquiring knowledge from medical staff, WeChat/micro-blog, or the internet were associated with a worse reproductive knowledge level. Senior high school or less educated women were 1.40 times more likely to have low RH knowledge scores than college or more educated women. Education was also found to have a powerful and proportional influence on RH knowledge in other studies [14, 15]. It is expected that less educated women are less likely to be aware of their health status and seek to improve their health knowledge. Furthermore, less educated women may have weaker decision-making power on health-related matters [14].

We found that women who had no history of gynecopathy had higher odds (2.06 times) of a lower RH knowledge level compared to women who had a positive history of gynecopathy. In agreement with our study, Liu and Zhang [15] also found that women without gynecological diseases had lower scores for RH knowledge than women with gynecological diseases, which may be attributed to the opportunity of women with gynecological diseases to receive relevant education from medical staff. Our findings also suggest that rural women who acquire RH knowledge from medical staff, WeChat/micro-blog, and the internet were all less likely to have a lower level of knowledge. Studies [28, 29] have shown that medical staff are the best way for women to acquire knowledge about RH. Medical staff should be encouraged to become the main providers of health education, to advertise and popularize women’s health knowledge, and to improve awareness of disease symptoms. This will promote the early detection, diagnosis, and treatment of diseases, thereby improving women’s overall health.

Other helpful knowledge sources in this study was the internet and social networks (WeChat/micro-blog). With the rapid development of information and communication technology, the internet and mobile phones have become the most important forms of interpersonal communication. A web-based study in rural China demonstrated that the use of a website to disseminate health information was not only feasible but that it also would be enthusiastically used by local health workers, teachers, and women’s groups [30]. We should develop more targeted interventions based on these technologies to provide rural women with more comprehensive and accurate RH knowledge.

Several limitations of the study are noted. Firstly, as the data were collected through questionnaires, reporting bias cannot be excluded. Secondly, self-reporting of gynecopathy is a poor measure for investigating prevalence. Thirdly, sampling was limited to a province in south-central China and most participants were of Han ancestry. In addition, there may have been some bias in the selection of counties and cities.

However, our study sample is large, and to our knowledge, this is the first study to examine the RH status and related knowledge of women aged 20–39 years in rural China. This study suggests that the RH status and knowledge level in this population are not optimal. Medical staff, social networks, and the internet are effective ways to provide RH knowledge to this population. This study can provide insight for future interventions to promote female RH in rural areas.

## Conclusion

There are many RH problems in women aged 20–39 years in rural China. Although the prevalence of gynecological diseases is not high, the rate of consultation for diseases is low, induced abortion rates are high, contraceptive methods are limited and misused, and RH related knowledge is seriously lacking.

Specific actions should be taken to create a health service program to popularize RH knowledge, reduce ineffective contraception and induced abortion, and increase the rate of disease consultation in this population. Medical staff, the internet, and social networks (WeChat/micro-blog) can all be effective interventions. Top-down initiatives should also be considered to monitor these efforts. Services should include health education and counseling, routine check-ups, and disease treatment to help women achieve healthy sex, pregnancies, and daily lives.

## Data Availability

The datasets generated during the current study are available from the corresponding author on reasonable request.

## Abbreviations

RH: Reproductive Health
WHO: World Health Organization
STIs: Sexually Transmitted Infections
RTIs: Reproductive Tract Infections
WRHQ: Women’s Reproductive Health Questionnaire
HPV: Human Papillomavirus
CNY: Chinese Yuan
USD: United States dollar
IUD: Intrauterine Devices
OC: Oral Contraceptives
